# Citrobacter koseri: A Cause of Silicone Oil Related Endophthalmitis after Post Pars Plana Vitrectomy

**DOI:** 10.1155/2023/3494521

**Published:** 2023-03-18

**Authors:** Luigi Sborgia, Valeria Albano, Giancarlo Sborgia, Francesco Boscia, Giovanni Alessio

**Affiliations:** ^1^Department of Basic Medical Sciences, Neurosciences and Sensory Organs, Eye Clinic, University of Bari, Piazza Giulio Cesare 11, 70124 Bari, Italy; ^2^Department of Basic Medical Sciences, Neurosciences and Sensory Organs, Eye Clinic, Piazza Giulio Cesare 11, 70124 Bari, Italy

## Abstract

**Purpose:**

We present a rare case of *Citrobacter koseri* culture-positive endophthalmitis in a postvitrectomy silicone oil-filled eye. *Case report.* A 64-year-old male patient presented to our ophthalmology emergency room with representative symptoms of acute endophthalmitis. He underwent a plana vitrectomy oil-filled tamponade previously. Preoperative and postoperative findings of the case were reported.

**Results:**

Culture tests of aqueous, silicone oil, and vitreous taps were positive for *Citrobacter koseri*.

**Conclusions:**

Culture-positive endophthalmitis in a silicone oil-filled eye has very rarely been in the literature. The described cases were caused by acute inflammatory reactions to silicone oil and were culture-negative. The postvitrectomy culture-positive endophthalmitis caused by *Citrobacter* is a very rare condition, and its management is not so smooth. Approaching with silicone oil removal, intraoperative intravitreal antibiotic injection, and silicone oil reinjection was performed in our case with good outcomes.

## 1. Introduction

Silicone oil has been commonly used as a long-term endotamponade agent in vitreoretinal surgery for several decades. It is used in the surgery of complex retinal detachment, proliferative retinopathy, viral, and bacterial retinitis [1]. Silicone oil is preferred as an endotamponade vitrectomy agent for infective endophthalmitis for its antimicrobial effects.

For these reasons, endophthalmitis associated with the silicone oil use are very rare, and its clinical findings and management are not well known [2].

We described a clinical presentation and management of a patient undergoing pars plana vitrectomy with oil-tamponade who referred to our ophthalmology emergency room with acute post-vitrectomy endophthalmitis culture-positive for *Citrobacter koseri*.

## 2. Case Presentation

**Figure 1:**
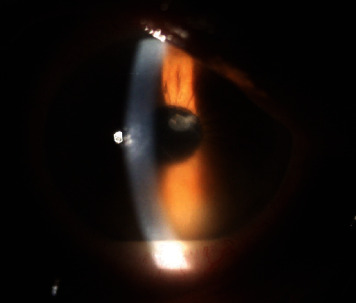
Slit-lamp photograph showing an edematous cornea with Descemet's membrane wrinkles; in the anterior chamber many small cells and a 2 mm hypopyon were found. A big fibrin flake near the IOL was well-revealed.

A 64-year-old male patient was referred to our emergency ophthalmic department with ocular ache and blurred vision in the right eye. He had a history of diabetes mellitus and hypertension. His past ocular medical history was significant for previous cataract surgery in both eyes and a pars plana vitrectomy for rhegmatogenous retinal detachment in diabetic retinopathy in 2021. In September 2021, he had a relapse of rhegmatogenous retinal detachment in diabetic retinopathy and underwent a pars plana vitrectomy with silicone oil and a 1000 cst injection. The entire retina is adhering through a silicone oil endotamponade. The postoperative visual acuity on the second day was 6/60. He received topical and steroid preparations. But after one week, I was presented with ocular pain and blurred vision in the right eye. His visual acuity deteriorated to hand motion. On the slit lamp examination, a severely injected conjunctiva was present, an edematous cornea with Descemet's membrane wrinkles and abundant keratic precipitates were appeared, the pupil was in middilated status and nonresponsive to light, the anterior chamber was occupied by cells and fibrin flakes near the intraocular lens (IOL), and hypopyon approximately 2 mm was detected. (Figure 1) The back structures, including the state of the lens, vitreous, and retina, were not well evaluated. A diagnosis of ocular inflammation due to post pars plana vitrectomy endophthalmitis was made. On day 1 of the clinical presentation, a sample of aqueous humor and vitreous was took under the local anesthesia, an antibiotic intravitreal injection of 1 mg/0,1 ml cefuroxime and 2.0 mg/0,1 ml ceftazidime was performed, and a subtenon injection of betamethasone was done. Microbiological analysis for cytology and culture for bacteria and fungi were done.

Reinforced cefazolin and reinforced tobramycin, atropine, and prednisone eye drops were administered every two hours in combination with systemic antibiotic and steroid treatment.

On 1 day after the intravitreal injection, the clinical symptoms are not better: the cornea was edematous, yet hypopyon was present. Only a decreased pain was revealed.

The patient underwent a new pars plana vitrectomy, wash-out of hypopyon from the anterior chamber, removal of IOL, silicone oil from the vitreous cavity, and silicone oil reinjection under local anesthesia. Samples of the anterior chamber, IOL, and silicon oil were sent to the lab for culture for bacteria and fungi.

The intraoperative pus in the vitreous and some retinal hemorrhages were detected.

At the end of the surgery, an intravitreal cocktail of vancomycin (1 mg/01 ml), ceftazidime (2.25 mg/0.1 mL), and amikacin 400 *μ*g/0.1 mL were injected in the vitreous cavity, according to the Endophthalmitis Vitrectomy Study Group (EVS) protocol, and Aprokam 0.1 mL was injected in the anterior chamber.

On day 1 after surgery, the visual acuity was light perception, and intraocular pressure was normal. A slight lamp evaluation revealed an edematous cornea with some Descemet's membrane wrinkles, fibrin flakes and small bodies in the anterior chamber, and hypopyon.

On day 2 after surgery, culture of the aqueous humor, IOL, and oil removed were positive for *Citrobacter koseri*.

*Citrobacter koseri* is proven resistant to various antibiotics while having high sensitivity to second- and third-generation cephalosporins. Therefore, the patient has carried on systemic medication with cephalosporins.

After one week from the surgery, the visual acuity improved to count fingers, the corneal edema restored completely, and the optic disc and retina were entirely visible at the fundus examination.

After 4 weeks, the best corrected visual acuity was improved to 6/60 (Snellen chart), with a refractive error of sf. +14.00 D, according to aphakic status and silicone oil injection.

## 3. Discussion

Endogenous endophthalmitis is an uncommon condition in healthy patients. [3] Frequently, it occurred in immunosuppressed individuals, often with severe comorbidities such as diabetes. In most cases, it originates from a spread from another infectious site, such as the liver, lung, kidney, and urinary system [4–6].

In our case, although the patient was affected by concomitant diabetes, no other evident site involved a part of the eye.

Post pars plana vitrectomy endophthalmitis under silicone-oil tamponade is a very rare condition. It has been reported to be 0,07% and 0,039%. [7] The majority of cases described in the literature were culture-negative, due to possible acute inflammatory reactions to silicone oil. [8].

Included studies, comprehensive of cases of endophthalmitis with silicone oil endotamponade, are summarized in Table 1 [9, 10].

Risk factors for post pars plana vitrectomy endophthalmitis occurrence, including sutureless surgery, wound leakage, hypotony, and vitreous incarceration in the wound [11].

Clinical progression of post pars plana vitrectomy endophthalmitis may be various; the diagnosis may be commonly confused with postoperative inflammation.

Gram-positive organisms were reported more frequently than Gram-negative ones: the first were found in 75–90% of cases, the second in only 6%. Although endophthalmitis caused by Gram-negative organisms is so rare, it is commonly associated with a negative visual prognosis [12].

Okonkowo et al. reported a case series of silicone oil-related endophthalmitis that was culture-positive for *Burkholderia crepacia*, belonging to Gram-negative organisms. [13].

*Citrobacter species* are very rare with respect to the other Gram-negative bacteria causing postoperative endophthalmitis.

*Citrobacter koseri* is an anaerobic, moving, Gram-negative bacteria belonging to the *Citrobacter species* (spp.), within the family Enterobacteriaceae. *Citrobacter koseri* is the most common among the *Citrobacter* spp., and it is very spread in nature, especially in water soil. Human infection is rare.

Likely other Gram-negative bacteria, endophthalmitis due to *Citrobacter koseri* may be related to poor visual results despite the clinical and surgical treatments. [14].

Although these cases have unclear management due to their rarity, they certainly need prompt intervention.

## Figures and Tables

**Table 1:** Included studies comprehensive of cases of endophthalmitis with silicone oil endotamponade.

	Type of study	*N* all eyes	N,Causatuve microrganism	Tamponade
Silpa Arca et al. (2021) [7]	Review	13	1, *Aspegillus flavus*	Silicone oil
Okonkwo et al. (2018) [9]	Case series	5	4, *Bukholderia cepacia*; 1, *Pseudomona aeruginosa*	Silicone oil
Steinmetz et al. (2018) [6]	Case series	2	ND	Silicone oil
Goel et al. (2015) [5]	Case report	1	1, *Pseudomonas aeruginosa*	Silicone oil
Roy (2013) [10]	Case series	5	3, *Citrobacter*	Silicone oil

*N*: number; ND: not determinable.

## Data Availability

The data are available from the Department of Basic Medical Sciences, Neurology and Sensory Organs, Institute of Ophthalmology, University of Bari. No restrictions on data access have been done.
